# Delineating the Molecular and Phenotypic Spectrum of the *CNGA3*-Related Cone Photoreceptor Disorder in Pakistani Families

**DOI:** 10.3390/genes13040617

**Published:** 2022-03-29

**Authors:** Sairah Yousaf, Nabeela Tariq, Zureesha Sajid, Shakeel A. Sheikh, Tasleem Kausar, Yar M. Waryah, Rehan S. Shaikh, Ali M. Waryah, Saumil Sethna, Saima Riazuddin, Zubair M. Ahmed

**Affiliations:** 1 Department of Otorhinolaryngology—Head & Neck Surgery, School of Medicine, University of Maryland, Baltimore, MD 21201, USA; sairayousaf61@gmail.com (S.Y.); saumilsethna@gmail.com (S.S.); sriazuddin@som.umaryland.edu (S.R.); 2 Department of Zoology, Sardar Bahadur Khan Women’s University, Quetta 81800, Pakistan; nabeela_trq@yahoo.com; 3 Institute of Molecular Biology and Biotechnology, Bahauddin Zakariya University, Multan 60000, Pakistan; zureeshasajid@gmail.com (Z.S.); rehansadiq80@yahoo.com (R.S.S.); 4 Molecular Biology and Genetics Department, Liaquat University of Medical and Health Sciences, Jamshoro 76090, Pakistan; compujin@gmail.com (S.A.S.); aliwaryah@lumhs.edu.pk (A.M.W.); 5 Department of Zoology, Government Sadiq College Women University, Bahawalpur 63100, Pakistan; tasleemkausar2008@hotmail.com; 6 Scientific Ophthalmic and Research Laboratory, Sindh Institute of Ophthalmology and Visual Sciences, Hyderabad 71000, Pakistan; yarmwaryah@hotmail.com; 7 Center for Applied Molecular Biology, University of the Punjab, Lahore 54500, Pakistan; 8 Department of Biochemistry and Molecular Biology, School of Medicine, University of Maryland, Baltimore, MD 21201, USA; 9 Department of Ophthalmology and Visual Sciences, School of Medicine, University of Maryland, Baltimore, MD 21201, USA

**Keywords:** achromatopsia, *CNGA3*, exome sequencing, missense variants, consanguineous families

## Abstract

Cone photoreceptor dysfunction represents a clinically heterogenous group of disorders characterized by nystagmus, photophobia, reduced central or color vision, and macular dystrophy. Here, we described the molecular findings and clinical manifestations of achromatopsia, a partial or total absence of color vision, co-segregating with three known missense variants of *CNGA3* in three large consanguineous Pakistani families. Fundus examination and optical coherence tomography (OCT) imaging revealed myopia, thin retina, retinal pigment epithelial cells loss at fovea/perifovea, and macular atrophy. Combination of Sanger and whole exome sequencing revealed three known homozygous missense variants (c.827A>G, p.(Asn276Ser); c.847C>T, p.(Arg283Trp); c.1279C>T, p.(Arg427Cys)) in *CNGA3*, the α-subunit of the cyclic nucleotide-gated cation channel in cone photoreceptor cells. All three variants are predicted to replace evolutionary conserved amino acids, and to be pathogenic by specific in silico programs, consistent with the observed altered membrane targeting of CNGA3 in heterologous cells. Insights from our study will facilitate counseling regarding the molecular and phenotypic landscape of *CNGA3*-related cone dystrophies.

## 1. Introduction

In vertebrates, visual perception is the consequence of the coordinative action of two basic phototransduction cascade’s, called cone and rod photoreceptor systems [1]. This retinal photoreceptor system’s first step is to absorb photons by their photopigments and mediate light signal translation into electric signal by the action of cyclic nucleotide-gated (CNG) channels [2]. CNG channels are found on plasma membranes of the outer segment of photoreceptor cells and are regulated by cyclic nucleotides, e.g., cGMP (cyclic Guanosine Monophosphate) and cAMP (cyclic Adenosine Monophosphate) [3]. Both cone and rod CNG channels are comprised of two structural subunits called A (CNGA1–4) and B subunits (CNGB1 and CNGB3) [4]. In the dark, CNG channels are present in an open conformation to maintain the depolarized state due to the presence of high cGMP levels. Upon light stimulation, a G-protein mediated cascade is initiated leading to cGMP hydrolysis and eventual CNG channel closure along with photoreceptor hyperpolarization [5]. As CNG channels are the main source for Ca^2+^ influx in cone and rod outer segments, channel closure results in decreased intracellular Ca^2+^ level that facilitates light response retrieval by phosphorylation of ocular pigments or regulation of guanylyl cyclase [6,7].

In cones, CNG channels are hetero-tetramers that are comprised of three CNGA3 subunits and one CNGB3 subunit [4]. Genetic variants of both *CNGA3* or *CNGB3* have been reported in humans with achromatopsia (ACHM), cone–rod dystrophy or other disorders such as progressive cone dystrophy [8,9,10,11]. Among *CNGA3* associated spectrum of inherited cone disorders, ACHM accounts for 20–30% cases and is characterized by vision impairment, partial or complete color blindness, nystagmus, and photo dysphoria especially during daytime [12]. Impaired or no CNGA3 protein synthesis may impact phototransduction by producing altered CNG channel stoichiometry that will either not bind with its ligand or have an increased sensitivity and hyperactivity with an ultimate consequence of photoreceptor dysfunction and degeneration [13]. Overall, an appropriate inter-subunit assembly, interaction and plasma membrane targeting is pivotal for normal CNG channel function. Here, we report molecular and clinical findings in three large Pakistani consanguineous families segregating ACHM phenotype due to biallelic variants of *CNGA3*.

## 2. Materials and Methods

### 2.1. Families’ Enrollment and Clinical Examination

The current study followed the tenets of the Declaration Helsinki and received approval from the Institutional review Board (IRB) Committees at the Bahauddin Zakariya University, Pakistan, Liaquat University of Medical & Health Sciences, Pakistan, and University of Maryland, USA. Two families (PKAB149, PKED06) were enrolled from the Punjab province of Pakistan, while the third family (LUSG06) was identified from the Sindh province. Informed written consent was obtained from all participants. Detailed ophthalmic examination including fundoscopy, slit lamp biomicroscopy, optical coherence tomography (OCT; TOPCON OCT 2000, Insight Eye Equipment, Tokyo, Japan) and electroretinography (ERG; RETeval, LKC Technologies, Inc., Gaithersburg, MD, USA) was performed at the local hospitals in Pakistan. Peripheral blood samples were collected from participating individuals for genomic DNA extraction and genetic analysis.

### 2.2. Mutation and Segregation Analysis

Sanger sequencing of all the coding exons and exon-intron junctions of *CNGA3* was performed using the proband DNA samples from families PKAB149 and PKED06. While whole exome sequencing (WES) was used to identify the disease-associated variants in family LUSG06. For WES, genomic libraries were recovered for exome enrichment using the Agilent Sure Select Human Expanded All Exon V5 (62 Mb) kit (Agilent corporation, Santa Clara, CA, USA). Libraries were sequenced on an Illumina HiSeq2500 (Agilent corporation, Santa Clara, CA, USA) with average 100× coverage. Data analysis used the Broad Institute’s Genome Analysis Toolkit [14]. Reads were aligned with the Illumina Chastity Filter with the Burrows-Wheeler Aligner [15], and variant sites were called using the GATK Unified Genotyper module. Selection of candidate disease associated variants was performed as described previously [16]. Sanger sequencing was used to confirm the segregation of the variants identified in all three families.

### 2.3. Bioinformatic Analysis

NCBI (National Center for Biotechnology Information) conserved domain was used to generate *CNGA3* protein structural domains, while Clustal omega multiple alignment was used to assess the conservation of the identified variants across several different species. Varsome (www.varsome.com, accessed on 15 October 2021) was used for pathogenicity predictions, and allele frequencies were obtained from gnomAD (https://gnomad.broadinstitute.org/, accessed on 15 October 2021) database. The 3-Dimensional (3D) protein structures were generated using Phyre2 server (http://www.sbg.bio.ic.ac.uk/phyre2/html/page.cgi?id=index, accessed on 15 October 2021) with “Intensive Mode” option, which is a combination of template-based modeling and ab initio methods.

### 2.4. Expression of Wild-Type and Mutant CNGA3 Channels

In order to determine the impact of the newly identified mutation on CNG channel functionality, homomeric as well as heteromeric CNG channels were heterologously expressed and surface expression quantified in human embryonic kidney 293 (HEK293) cells, as previously described [17].

## 3. Results

### 3.1. Family PKAB149

**Figure 1:**
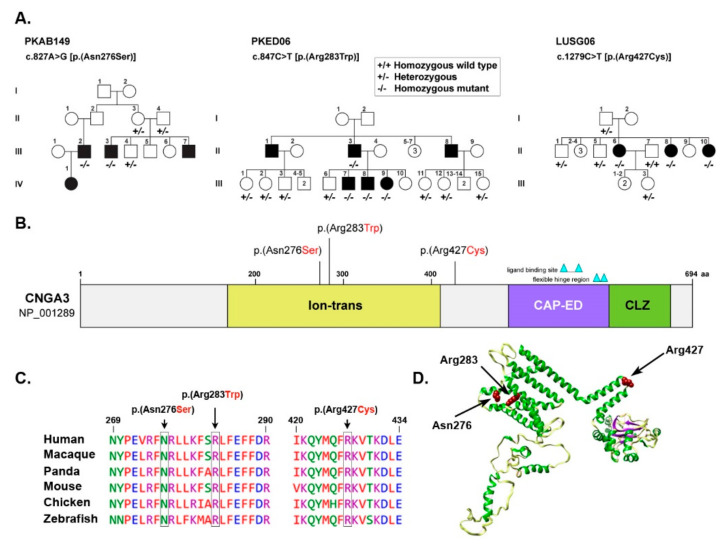
(**A**) Pedigrees of Pakistani families segregating with *CNGA3* variants. Filled and empty symbols represent affected and unaffected, respectively. The genotypes are mentioned below each subject, enrolled in the study. (**B**) Graphical representation of the human *CNGA3* protein domain, the location of identified variants is also marked. (**C**) Clustal alignment of CNGA3 protein variants identified in the study. (**D**) The 3-dimensional representation of CNGA3, generated by Phyre 2 and visualized by Chimera. The residues of interest are shown in brick red color; however, secondary structure of the protein is shown in the following colors: Helix, green; strands, purple and coils light yellow. Hydrogen banding pattern is also shown in light pink threads.

**Figure 2:**
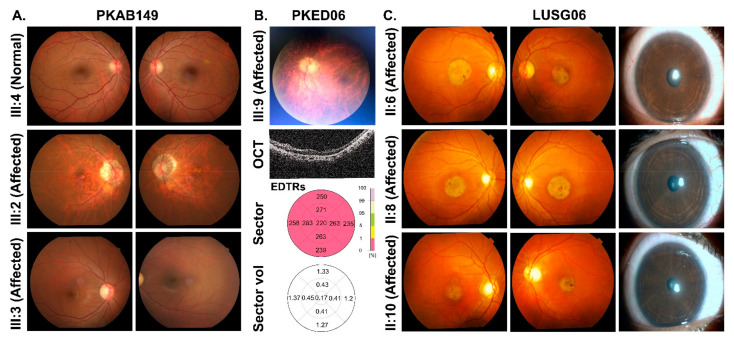
(**A**) Fundoscopy images of family PKAB149. (**B**) Representative image of one of the affected individuals of family PKED06 along with its OCT (middle panel) and retinal thickness map (bottom panel), showing average of retinal thickness measurements. (**C**) Fundoscopic (left) and slit lamp (right) images of affected individuals of family LUSG06.

**Figure 3:**
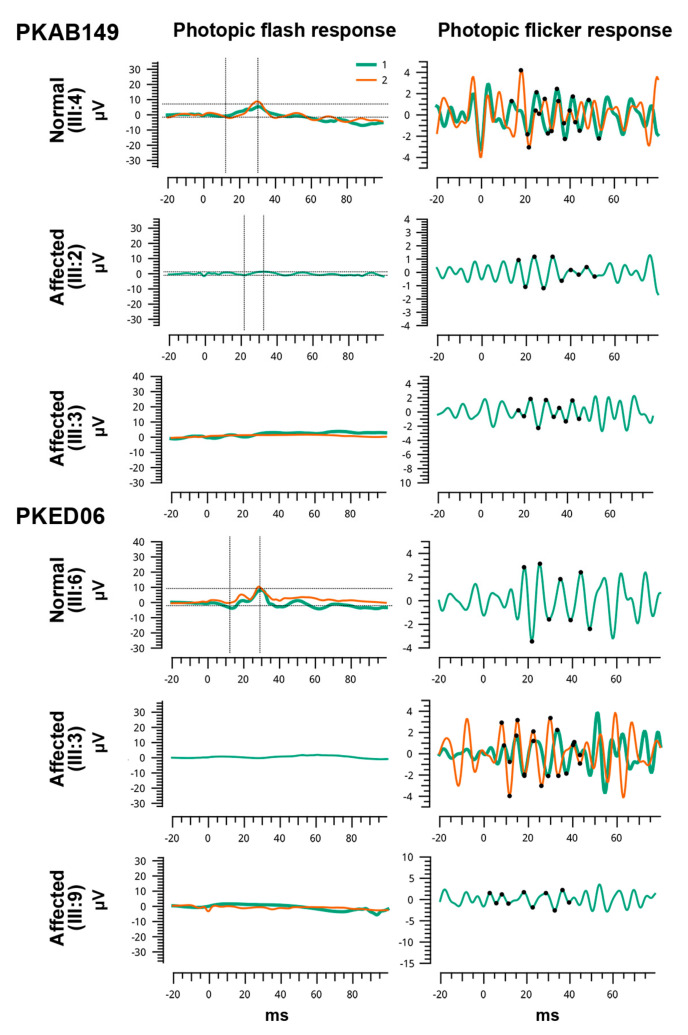
Shown are the full-field ERG photopic responses of two normal and four affected individuals homozygous for *CNGA3* identified known missense variants. In order to show reproducibility, two responses (green and orange) have been superimposed for each ERG condition. ERG responses of affected individuals of both families revealed severe reduction in photopic amplitudes.

A multi-generation family with four affected individuals was ascertained from Punjab province of Pakistan. Only two (III:2 and III:3) of the four affected individuals participated in the study (Figure 1A). According to family history interviews, both participating affected individuals had photophobia, nystagmus, and lack of color discrimination since childhood. They experienced intense uneasiness during daylight with normal night vision. Ophthalmic examination showed reduced visual acuity of 2/60 for both eyes of affected individual III:2, while 6/60 in OD (oculus dextrus) and 6/36 in OS (oculus sinister) of affected individual III:3 (Table 1). Individual III:2 had small atrophic macular lesions, myopic appearing fundus with retinal thinning, mild myopic astigmatism, and bilateral peripapillary atrophy (Figure 2A). However, his cousin, affected individual III:3, had relatively normal appearing fundus with mild myopic astigmatism (Figure 2A). Photopic ERG responses were severely reduced in the two evaluated affected individuals (Figure 3).

Sanger sequencing of all the exons of *CNGA3* (NM_001298), revealed a transition variant c.827A>G, predicted to introduce a missense change in the encoded protein (p.(Asn276Ser)), segregating in a homozygous fashion among the affected individuals (Figure 1A). The p.(Asn276Ser) change is present in the ion transport domain of CNGA3 (Figure 1B), which has an essential role in maintaining ionic balance. The asparagine residue at position 276 is highly conserved across several different species (Figure 1C), and replacement with serine (p.(Asn276Ser)) is predicted to be pathogenic by several in silico prediction programs (Table 2). Our 3D molecular modeling prediction indicated that replacing asparagine with small sized serine at amino acid position 276 might result in hydrophobicity differences and possible loss of external interactions (Figure 1D).

### 3.2. Family PKED06

A large consanguineous Pakistani family (PKED06), including six affected individuals suffering with pendular nystagmus, photophobia (in early infancy) with myopia (Table 1), was enrolled from Punjab province of Pakistan. Myopic appearing fundus with retinal thinning and peripapillary optic atrophy was observed in affected IV:9 (Figure 2B). Similarly, OCT also revealed retinal thinning throughout, particularly of the outer nuclear layer (ONL), and otherwise normal appearing foveal architecture. A disruption of inner/outer segment junctional complex with RPE (retinal pigment epithelium) thinning may indicate diffuse chorioretinal atrophy (Figure 2B). Axial scan of right eye of affected IV:9 showed intact posterior capsule region (Figure 2B). In transverse and longitudinal scan, right eye presented multiple low intensity echoes in vitreous cavity, while retina was in situ (Supplementary Materials, Figure S1). Choroid, optic nerve head, extra-ocular muscles and retroocular fat were within the normal range.

Through candidate gene screening, a homozygous transition variant c.847C>T (p.(Arg283Trp)) in *CNGA3* was found segregating with the phenotype in the family PKED06 (Figure 1A). The p.(Arg283Trp) missense variant is also located within the ion transport domain (Figure 1B) and predicted to be pathogenic with CADD (Combined Annotation Dependent Depletion) score of 27 (Table 2). The arginine residue at position 283 is also evolutionary conserved (Figure 1C). According to HOPE (Have (y)Our Protein Explained) prediction program, the replacement of arginine at position 283 with tryptophan results in disturbed ionic interaction due to large size of tryptophan, no charge, and more hydrophobic nature as compared to wild type arginine residue. Arginine is predicted to form a salt bridge with aspartic acid and glutamic acid at positions 217 and 286, respectively, which are lost due to replacement with tryptophan (Figure 1D), and hence, could distort protein topology and results in misfolded protein.

### 3.3. Family LUSG06

**Figure 4:**
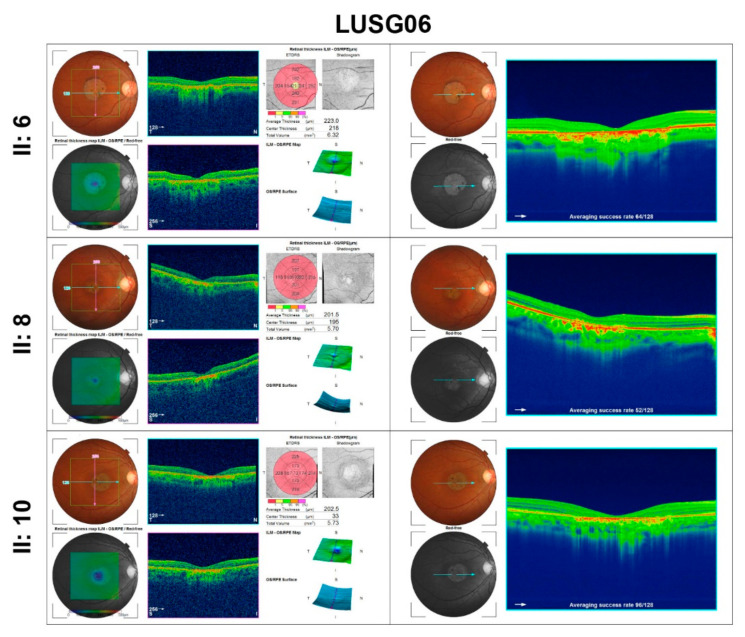
Optical Coherence Tomography (OCT) images of macula of family LUSG06 along with retinal thickness.

In family LUSG06, who were enrolled from Sindh province of Pakistan, the funduscopic examination of the affected individuals revealed the presence of bilateral symmetric pallor optic discs (Figure 2C). Affected individuals II:6, II: 8, and II:10 had mild visual acuity loss and minimal refractive error (Table 1), bilateral atrophic macular lesions 1–1.5-disc diameters on fundus examination, with normal optic nerve, vessels, and anterior segment (Figure 2C). OCT imaging of their eyes was consistent with outer segment thinning, inner retinal layer remodeling, and RPE loss at the fovea and perifovea, and mild retinal thinning with otherwise normal architecture in the perimacular region (Figure 4). ERG photopic responses were reduced in the two evaluated affected individuals (Figure 3), while all affected individuals had no color vision.

Whole exome sequencing of proband (individual III:6) revealed a transition variant c.1279C>T (p.(Arg427Cys)) in exon 8 of *CNGA3*, which segregated with the phenotype in the family LUSG06 (Figure 1A). The p.(Arg427Cys) allele had a very low allele frequency (MAF: 0.0003874) in gnomAD database and was predicted pathogenic by multiple pathogenicity prediction programs (Table 2). In contrast to the above-mentioned variants, the p.(Arg427Cys) variant is located in the sixth topological domain of CNGA3 (Figure 1B) facing extracellular surface. The arginine residue at position 427 is highly conserved across several different species (Figure 1C). The p.Arg427 residue is positively charged and predicted to form a salt bridge with lysine at position 692. The replacement of p.Arg427 with a smaller, neutral residue, cysteine (p.Arg427Cys) might result in the loss or disturbance of this ionic interaction leading to impaired protein and faulty biological function (Figure 1D).

### 3.4. The p.Asn276Ser Variant Affects the Integration of the CNGA3 Protein into Plasma Membrane

**Figure 5:**
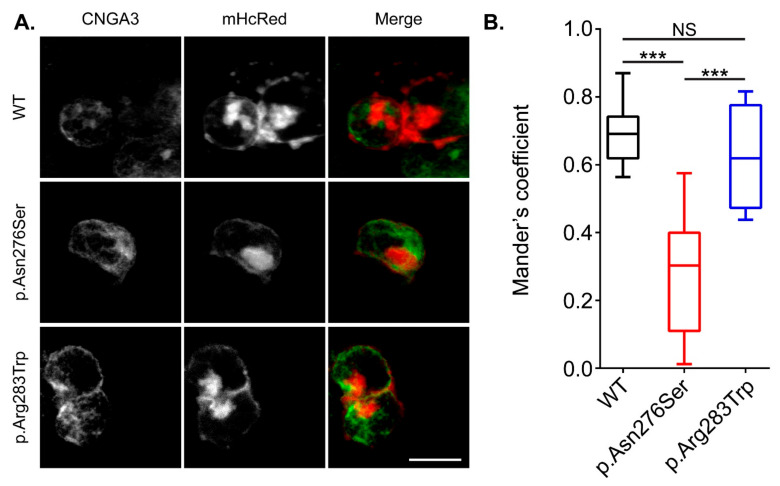
(**A**) Surface expression study of wild type (WT) and mutant CNGA3 protein. CNGA3 is labeled in green; however, mHcRed (red) is used to mark plasma membrane. (**B**) Graphical representation of Manders’ coefficient used to quantify colocalization of green and red fluorescence at the plasma membrane in 20 transfected cells per construct (*** *p* < 0.001). NS, not significant.

Previous studies have shown decreased CNGA3 channel density in the plasma membrane due to p.Arg427Cys variant [21]. To determine if p.Asn276Ser and p.Arg283Trp variants also have similar impact, we expressed *CNGA3* channels harboring these two variants in HEK293 cells and performed immunocytochemistry experiments (Figure 5A). In our study, HEK293 cells were co-transfected with a plasmid encoding a red-fluorescent protein (mHcRed) targeted to the plasma membrane. To quantify the extent of co-localization of the CNGA3 mutant channels with the plasma membrane, we calculated the Manders’ overlap coefficient [22]. Although the p.Arg283Trp variant did not impact the membrane targeting of CNGA3 channel in comparison with the wild-type protein, however, the homomeric CNGA3 with p.Asn276Ser variant showed a significantly reduced Manders’ overlap coefficient, indicating that the integration of CNGA3 channel into the plasma membrane was reduced due to impaired protein folding and/or trafficking (Figure 5B). In contrast, p.Arg283Trp variant did not impact the membrane targeting of CNGA3 channel (Figure 5B).

## 4. Discussion

In humans, cGMP (second messenger) mediates G-protein coupled cascade to carry out visual signal transduction. CNGA3 protein is activated by this cGMP and triggers cation channel opening which ultimately lead towards depolarization of cone photoreceptors. In this study, we have reported three large unrelated consanguineous Pakistani families segregating with three known *CNGA3* substitution alleles in an autosomal recessive manner. Clinical findings of the presented patients include outer nuclear layer thinning with or without macular atrophy, myopic fundus, nystagmus, and photophobia highlighting the role of *CNGA3* in normal vision. Previously, fundus examination and OCT findings of *CNGA3* variants revealed age-dependent foveal hyperfluorescence patterns in achromatopsia affected young patients in comparison to older patients who have foveal atrophy and outer retinal cavitation along with unique hypo/hyperfluorescence [23]. Along with rod monochromacy, a wide range of *CNGA3* variants are also reported to cause cone-dominant retinal malformation in autosomal recessive manner. For instance, achromatopsia, cone or cone-rod dystrophy, Leber congenital amaurosis, and Oligo trichromacy [24]. A cone–rod degeneration phenotype has been noted in Pakistani patients previously, in a large family segregating *CNGA3* (p.(Cys319Arg)) in an autosomal recessive pattern of inheritance [17]. *CNGA3* takes part in forming cone’s ion-conduction channel with its modulator called *CNGB3*.

To date, there are about 197 different variants reported for *CNGA3* responsible to cause different disease phenotypes, e.g., achromatopsia, cone–rod dystrophy, and retinitis pigmentosa (HGMD) amongst others. Additionally, *CNGB3* mutations in human can cause severe reduction in rod function [25,26]. Two of the three identified variants are the part of ion transport domain (p.(Asn276Ser), p.(Arg283Trp)) of CNGA3 protein that help in maintaining ionic balance. Immunocytochemistry studies in heterologous cells revealed an altered surface expression for CNGA3 harboring p.Arg276Ser variant, which could be due to misfolding of the encoded protein. Misfolded proteins undergo degradation within endoplasmic reticulum before reaching their targeted destinations. A recent study has shown 1.5- to 4.8-fold upsurge in the surface countenance of *CNGA3* harboring missense variants after treating the heterologous cells with chemical and pharmacological chaperons [27], which could be further developed into potential therapeutic drugs for individuals inheriting *CNGA3* disease causing variants.

Adeno-associated virus-mediated gene replacement therapy is also being developed as another treatment option for *CNGA3* patients. Previous studies have shown that loss of *CNGA3* function leads to rod dysfunction, potentially following alterations in cone synapses [28]. *Cnga3* null mice also exhibit loss of M-cone opsin-expressing photoreceptors, which can be rescued by gene replacement packaged into adeno-associated virus [29]. Intriguingly, a recent study reported outcome of a 3-year retinal gene therapy trial (AAV8.*CNGA3*) for nine patients affected with *CNGA3*-associated achromatopsia [30]. In this human clinical trial, no serious adverse effects of the treatment at year 1 were noted, while significant improvement in functional benefits when compared with untreated eye, which persisted in year 2 and 3 as well [30]. Thus, exploring the genetic causes of retinal dysfunction in humans not only helps in understanding disease pathology, natural history, genotype-phenotype correlations, but now could also enlist them for ongoing clinical trials and other innovative treatment modalities.

## Figures and Tables

**Table 1:** Clinical findings of Pakistani families segregating recessively inherited achromatopsia.

Family ID	Individual ID	Phenotype	Age (Year)	Visual Acuity		Refractive Error (Diopter)	Fundus	Photophobia	Nystagmus
				OD	OS				
PKAB149	III:2	Affected	40	2/60	2/60	−2.25/−2.75*44 −4.00/−2.25*126	Abnormal	Yes	Yes
	III:3	Affected	30	6/60	6/36	−3.75/−2.25*10 −1.50/−2.25*1	Abnormal	Yes	Yes
	III:4	Normal	42	6/9	6/9	+0.25/−0.75*12 +0.5/−0.5*15	Normal	No	Yes
PKED06	III:9	Affected	35	2/60	2/60	−9.00*149 −3.25	Abnormal	NA	NA
LUSG06	II:6	Affected	24	6/36	6/36	+0.25/−0.75*105 +1.50	Abnormal	NA	NA
	II:8	Affected	21	6/36	6/36	+0.25/−2.75*175 −2.50/−2.75*160	Abnormal	NA	NA
	II:10	Affected	22	6/36	6/36	−0.75/0.75*100 −0.25/−1*65	Abnormal	NA	NA

NA: Not Available.

**Table 2:** Pathogenicity predictions of *CNGA3* variants identified in Pakistani families.

Family ID	PKAB149	PKED06	LUSG06
Gene	*CNGA3*		
Nucleotide change	c.827A>G	c.847C>T	c.1279C>T
Amino acid change	p.(Asn276Ser)	p.(Arg283Trp)	p.(Arg427Cys)
gnomAD	Not found	0.0001056	0.0003874
CADD	25	27	32
SIFT (Sorting Intolerant From Tolerant)	Damaging	Damaging	Damaging
Polyphen2	Probably damaging	Probably damaging	Probably damaging
MutationTaster	Disease causing	Disease causing	Disease causing
FATHMM-MKL	Damaging	Neutral	Damaging
MutationAssessor	High	High	High
MetaSVM	Damaging	Damaging	Damaging
MetalR	Damaging	Damaging	Damaging
Reference	[18]	[19]	[20]

## Data Availability

The variants found in this study are available in the ClinVar database.

## Supplementary Materials

The following supporting information can be downloaded at: www.mdpi.com/article/10.3390/genes13040617/s1, Figure S1: Axial B-scan of right eye of affected individual (III:9) of PKED06.
