# Supplementary material for: Delineating the Molecular and Phenotypic Spectrum of the CNGA3-Related Cone Photoreceptor Disorder in Pakistani Families
Source: Genes (Basel). 2022 Mar 29;13(4):617. doi: 10.3390/genes13040617 (PMC9031457; doi:10.3390/genes13040617)
Supplement: Supplementary file 1 [file genes-13-00617-s001.zip › genes-1600345-supplementary.pdf]

Delineating the Molecular and Phenotypic Spectrum of the *CNGA3*-Related Cone Photoreceptor Disorder in Pakistani Families

Sairah Yousaf [1], Nabeela Tariq [2], Zureesha Sajid [3], Shakeel A. Sheikh [4], Tasleem Kausar [5], Yar M. Waryah [6], Rehan S. Shaikh [3,7], Ali M. Waryah [4], Saumil Sethna [1,†], Saima Riazuddin [1,8] and Zubair M. Ahmed [1,8,9,*]

B scan

Affected (III:9)

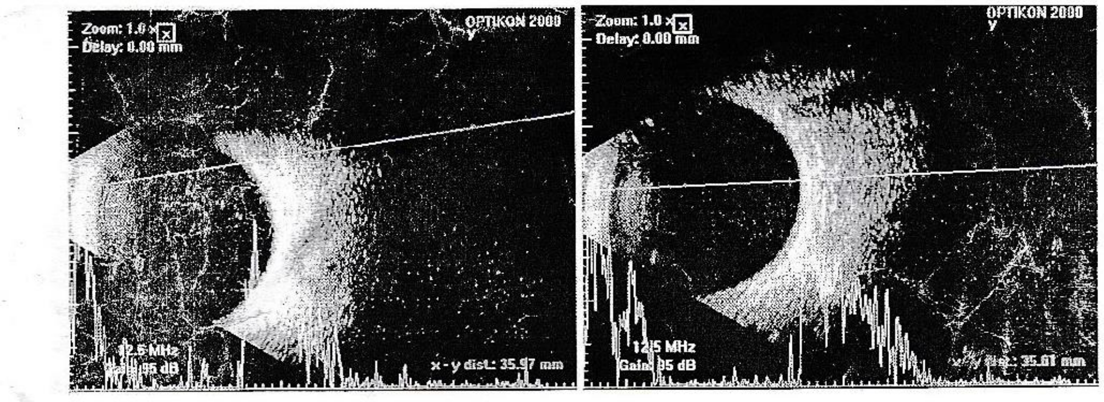

Axial scan of right eye shows intact **posterior capsule region.** In transverse and longitudinal scan right eye shows **multiple low intensity echoes in vitreous cavity. Retina is in situ.** Choroid, Optic nerve head, extra-ocular muscles and retro-ocular fat are within normal limits. No evidence of IOFB is seen.

IMPRESSION: R.E vitreous opacities

Figure S1: Axial B-scan of right eye of affected individual (III:9) of PKED06.
